# Comprehensive Genomic Analysis of Trihelix Transcription Factor Genes and Their Expression Underlying Abiotic Stress in Euphrates Poplar (*Populus euphratica*)

**DOI:** 10.3390/plants14050662

**Published:** 2025-02-21

**Authors:** Boniface Ndayambaza, Jianhua Si, Xin Zhao, Yingxue Zhao, Dongmeng Zhou, Bing Jia, Xinglin Zhu, Zijin Liu, Xue Bai, Boyang Wang

**Affiliations:** 1 Key Laboratory of Ecohydrology of Inland River Basin, Northwest Institute of Eco-Environment and Resources, Chinese Academy of Sciences, Lanzhou 730000, China; ndayambazab78@mails.ucas.ac.cn (B.N.); zhoudongmeng@nieer.ac.cn (D.Z.); jiab@lzb.ac.cn (B.J.); zxinglin@yeah.net (X.Z.); liuzijin@nieer.ac.cn (Z.L.); baixue@nieer.ac.cn (X.B.); wangboyang23@mails.ucas.ac.cn (B.W.); 2 University of Chinese Academy of Sciences, Beijing 100049, China; 3 Institutional Center for Shared Technologies and Facilities of NIEER, Chinese Academy of Sciences, Lanzhou 730000, China; zhaox@lzb.ac.cn (X.Z.); yingxuezhao1122@163.com (Y.Z.)

**Keywords:** comprehensive analysis, TTF family genes, *Populus euphratica*, salt stress, drought stress, expression pattern

## Abstract

Trihelix transcription factors (TTFs) are light-sensitive proteins characterized by a triple-helix structure that play a crucial role in regulating plant growth and development, especially in response to abiotic stressors, such as drought and salinity. This intriguing family of proteins has been the focus of extensive functional studies across various plant species. Despite their recognized significance, the trihelix family in *Populus euphratica* has not been thoroughly explored, warranting more attention. This study identifies 35 full-length trihelix genes in *Populus euphratica*, which are grouped into five categories (GT-1, GT-γ, GT-2, SIP1, and SH4) based on their conserved motifs and structural similarities, and these genes are unevenly distributed across 19 linkage groups on the chromosomes. A syntenic analysis was conducted in *P. euphratica*, comparing it to various other species. The promoters of *P. euphratica* contain numerous stress-responsive cis-elements, indicating the potential for these trihelix genes to respond to abiotic stress. RT-qPCR analysis discovered significant induction of the trihelix gene family in response to drought and salt stress, with 21 *PeuTTF* genes exhibiting distinct expression levels under drought conditions and five *PeuTTF* genes responsive to salt stress. Notably, heightened expression of *PeuTTF6*, *PeuTTF9*, and *PeuTTF20* was observed in both roots and leaves during drought stress, suggesting that TTF expression is connected to the plant’s response to such conditions. Additionally, significant increases in expression were noted for *PeuTTF2*, *PeuTTF31*, and *PeuTTF32*, which may be convoluted in the response to salt stress. These discoveries highlight the role that *PeuTTF* genes play in improving drought tolerance in *P. euphratica* plants. We offer new perspectives on the evolutionary trends and variants of *PeuTTF* genes in *P. euphratica*, and we establish the groundwork for understanding the functional properties of *PeuTTF* genes under salt-stressed and drought-stressed conditions. This study provides opportunities for the advancement of desert poplar agriculture and may have wider ramifications for tree plant breeding techniques targeted at improving tree performance and durability, particularly in dry areas.

## 1. Introduction

Woody plants are significantly impacted by abiotic factors, like salinity, drought, and high temperatures as well as real-time environmental changes. This results in poor growth and lower yields of forest trees [1]. Worldwide, further severe abiotic stress has been forecasted in arid and semi-arid regions due to climate change [2]. Deserts partake particularly of the severest ecosystems since they conglomerate high temperatures and low rainfall. Globally, these stressors embrace heavy metals, high pH, salinity, extreme temperatures (hot and cold), and risky water levels (drought) [3]. In order to protect themselves from biotic and abiotic stress, plants have evolved natural defenses, such as shutting stomata, lowering transpiration, generating ABA, and storing H_2_O_2_ [4]. Plant development and growth are impacted by drought and salt stress, which also affects soil microbiology and plants [5]. Plants frequently encounter these two stressors (drought and salinity), which inhibit biomass formation and plant growth. Because *P. euphratica* can persist in highly arid desert conditions, it is considered a crucial model plant for studying the responses of woody plants to abiotic stressors [6]. The *P. euphratica* species is primarily dispersed in Southwestern Europe, Western Asian countries, Central Asia, China’s western Inner Mongolia, Xinjiang, and other various arid regions. It also originated in numerous other countries outside of Asia, like the Middle East and North Africa [7]. The distribution of *P. euphratica* is predominantly found in the arid and semi-arid regions of China, particularly along riverbanks and desert margins in areas such as Inner Mongolia. While *P. euphratica* provides certain ecological benefits, its overexpansion in semi-arid areas of China, particularly in Inner Mongolia, can lead to negative consequences, such as increased soil salinity, which adversely affects surrounding flora [8]. Also, its dense root systems may compete with native species for water, potentially reducing biodiversity. Likewise, excessive growth can alter local water tables, disrupting traditional land use practices and impacting agricultural productivity [9]. Breeding programs continue to deliver sustainable increases in poplar tree yields [10]. However, although research has been conducted on *P. euphratica*, biotechnology research is still lacking on this species, especially in arid regions, which will boost the investigation of the role of *P. euphratica* in these arid and semi-arid environments, focusing on its impact on soil salinization, water retention, and biodiversity. By understanding how this tree species adapts to and modifies its harsh surroundings, we can gain insights into its potential to mitigate desertification, enhance ecosystem resilience, and improve land management practices in arid and semi-arid zones.

The poplar tree is crucial for pulp and biofuel production, has seen increased research due to its ecological and timber wood, plywood, domestic tourism, and raw materials for paper industry significance [11]. *P. euphratica* trees have developed defense mechanisms against adverse conditions. They have a distinct leaf heteroblasty pattern and are found in desert oases [12,13]. To overcome abiotic stressors, poplar plants have made morphological, physiological, and biochemical adaptations [14,15]. For instance, the fact that *P. euphratica* can adapt to high salt levels and high pH could be because of its natural ability to handle drought [16]. Stress-responsive genes are associated with proteins and enzymes that regulate ion homeostasis, maintain cell membrane integrity, and detoxify reactive oxygen species [17,18]. Consequently, *P. euphratica*, as the dominant tree species of desert riparian forests along the central Asia inland rivers in arid areas, plays an important role in maintaining the riparian ecosystems [19,20]. Scientific inquiry primarily focuses on understanding the mechanisms that enable drought and salt tolerance of poplar species in arid environments. Additionally, researchers aim to identify genes associated with the response to these stressors—drought and salt stress. Intriguingly, transcription factors (TFs) play a key role in signal transduction, signal perception, and the transcriptional control of abiotic stress. Nonetheless, the trihelix genes present in poplar species remain underexplored, highlighting the need for comprehensive research in arid areas.

Trihelix transcription factors (TTFs) are essential proteins that play a critical role in various aspects of plant growth and development. These factors are involved in light regulation and morphogenesis, influencing traits, such as trichomes, stomata, and flower formation. Additionally, TTFs are vital during late embryogenesis and help plants respond to environmental stressors. TTFs belong to a specific family of plant transcription factors characterized by their unique DNA-binding structural domain, which features three tandem helixes arranged in a helix-loop-helix-loop-helix configuration. This distinctive structure enables TTFs to interact effectively with DNA sequences. Furthermore, they are also referred to as the GT factor family, as their DNA-binding domain specifically recognizes the GT motif, which includes a light-responsive element crucial for plant adaptation to changing light conditions [21]. TTF proteins typically have various domains in either the N- or C-terminus [22]. These domains have exceedingly conserved protein sequences and exist generally consistently across various member families. They are shared by five subgroups in *Arabidopsis thaliana*: GT-1, GT-2, GTγ, SH4, and SIP1 [23]. The conserved domain of the trihelix transcription factor coincides with the helix-turned-angle helix structure of the MYB transcription factor, encompassing the key characteristics of the MYB transcription factor. These variations could be associated with target gene sequence binding and functional disparities among altered subfamily genes. TTF proteins, present in plants, are also found in the intestinal cavities of animals and insects [21]. TTFs are primarily investigated in plants, where most species have a small family consisting of 30 to 60 members, while individual polyploid species can have around 100. For example, *Arabidopsis thaliana* has been identified to have 30 members within the trihelix family [24], 56 in *Populus trichocarpa* [25], 56 in *Osmanthus*. *fragrans* [26], 96 in *Solanum lycopersicum* [27], 94 in *Triticum aestivum* [28], 41 in *Oryza sativa* [29], 40 in *Sorghum bicolor* L. Moench [30], 35 in *Phyllostachys edulis* [31], and 102, 51, and 51 in *Gossypium hirsutum* (AD)1, *Gossypium raimondii* (D5), and *Gossypium arboreum* (A2), three cotton species, respectively [32]. GTL1 is a transcription repressor that promotes stomatal density and regulates cell cycle genes. Previous research confirms their role in plant development, such as *AtGT-3b* activation under salt stress, which may interact with the GT-1 cis-element of the SCaM-4 promoter in *G. max* [33]. GT-2 LIKE 1 (*AtGTL1*) enhances drought tolerance and water use efficiency by trans-repressing STOMATAL DENSITY AND DISTRIBUTION1 gene expression, which regulates stomatal production in *A. thaliana* [34]. The analogous *AtGTL1* gene, *TaGT2L1D*, also impacts the development of floral organs in wheat and has a similar function [35]. Three GTγ clade genes (*OsGTγ-1*, *OsGTγ-2*, and *OsGTγ-3*) showed considerably higher expression levels in rice during salt stress; additionally, the *OsGTγ-1* gene responded to both drought and cold stress [26]. In the process of rice seed shattering, activation of cell separation is facilitated by the Shattering1 (SHA1) gene, which codes for a member of the trihelix SH4 subfamily [36]. In addition to being important for plant growth and development, trihelix genes are also involved in the biotic and abiotic stress responses of plants, such as defense mechanisms triggered by pathogens and adaptations to drought, salt, and cold [24,37]. For instance, trihelix genes regulate cold stress response, with overexpression of GT-1 gene *ShCIGT* enhancing tomato’s cold and drought tolerance [38], and transgenic *A. thaliana* plants showing strong freezing stress resistance [39]. BnSIP1-1 protein expression, induced by salt and osmotic stress, significantly increases the germination rate in oilseed rape seeds, highlighting its role in ABA synthesis and signaling [40].

This study analyzed the trihelix transcription factor gene family (*PeuTTF*) in *P. euphratica* to understand its roles in responding to salt and drought stress. Despite being homologous genes from different poplar species, their expression patterns differ under these stressors, and little is known about TTFs in *P. euphratica*. We identified 35 PeuTTF candidate genes by referencing the TTF gene family of *P. trichocarpa*. We projected and examined the chromosomal location, gene expansion, gene structure, and promoter cis-acting elements of potential TTF genes. Additionally, predictions and analyses were performed about the physiochemical properties, like molecular weight (MW), isoelectric point (pI), instability index, and GRAVY index; subcellular localization; motifs; and evolutionary relationships of the proteins that they encrypt. We also examined gene responses to salt and drought stress across various tissues, including leaves, stems, and roots. This study aimed to comprehensively analyze the TTF gene family in *P. euphratica* species in arid regions. So far, we have studied the expression pattern of selected *P. euphratica* trihelix genes underlying the response to abiotic stress, including salt and drought. Our findings suggest that *PeuTTF6*, *PeuTTF9*, and *PeuTTF20* may be key indicators of TTF expression in response to drought stress. Furthermore, there was an increase in the expression of *PeuTTF2*, *PeuTTF31*, and *PeuTTF32*, which may respond to salt stress. These genes have significant implications for breeding programs aimed at improving poplar resilience in dry environments. They also help in understanding the adaptive evolution of *P. euphratica* in drought desert areas. Comprehensions into the roles of trihelix genes in the poplar response to abiotic stressors (drought and salt) are offered by our early findings, which will facilitate further research in arid regions.

## 2. Results

### 2.1. Identification of the Trihelix Transcription Factor Genes in PeuTTF Proteins

Using the Phytozome V13 (https://phytozome-next.jgi.doe.gov/, accessed on 6 June 2024) and NCBI databases (https://www.ncbi.nlm.nih.gov/protein, accessed on 6 June 2024), trihelix genes in the genome of *P. euphratica* were identified. We eventually discovered 35 non-redundant TTF genes with the Pfam domain (PF13837) from the *P. euphratica* genome database after eliminating repetitive and redundant genes. Compared to *Arabidopsis*, *P. euphratica* has a much greater number of trihelix genes (30) [24] and is lower in number than *P. trichocarpa* (56) [25]. The corresponding amino acid sequences of these genes matched the ASIL1/2-like (IPR044823) domain and possessed the MYB_DNA-binding domain (IPR044822), as illustrated in Supplementary Table S1. The ASIL1/2-like domain was present in 9 of the 35 PeuTTF proteins, while the MYB_DNA-binding domain was present in all of the genes. These discovered TTF proteins (PeuTTF1 to PeuTTF35) were given names based on where they were found on the chromosome and how many TTFs were found on each chromosome—between one (Chr 1) and nineteen (Chr 19). The *PeuTTF* gene information is displayed in Table S1 along with the gene identities, locations, gene lengths, and CDS lengths. The lengths of the CDSs varied from 753 bp (*PeuTTF35*) to 2430 bp (*PeuTTF6*), with an average length of 1311.3 bp. Similarly, the predicted protein lengths varied from 250 aa (PeuTTF35) to 809 aa (PeuTTF6), with an average length of 436.1 aa. The range of projected molecular weights ranged from 27,683.74 Da for PeuTTF9 to 88,363.82 Da for PeuTTF6. The majority of PeuTTF protein products had pI values between 4.45 and 9.64, with all of them being below 10 (Supplementary Table S1(a&b)).

### 2.2. Phylogenetic Tree and Gene Structure Analysis, Chromosome Distribution, and Gene Duplication in PeuTTF Proteins

**Figure 1:**
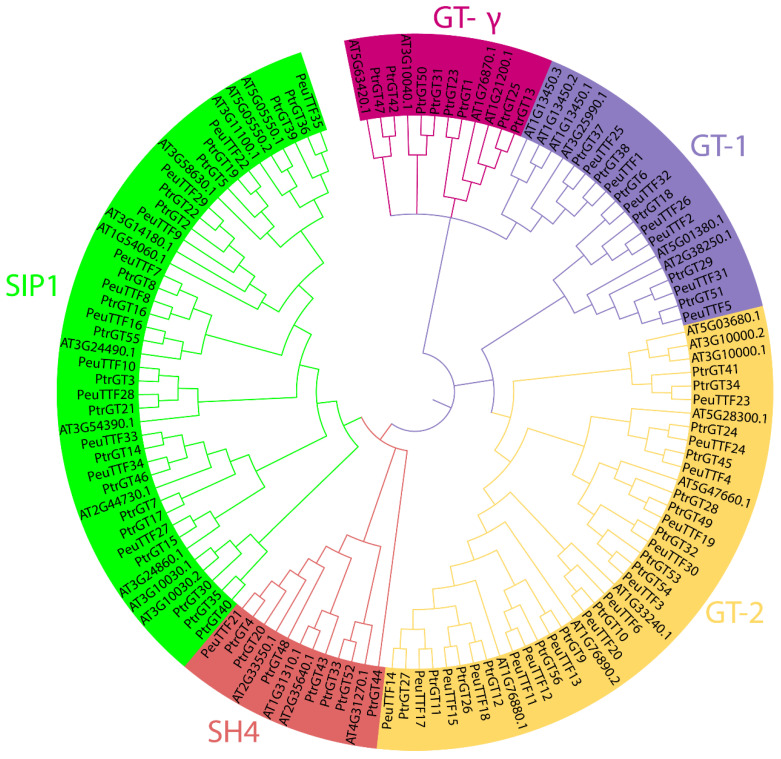
Phylogenetic analysis of TTF proteins from *P. euphratica*, *P. trichocarpa*, and *A. thaliana* was conducted. The complete amino acid sequences of 35 PeuTTF proteins, 56 PtTTF proteins, and 34 AtTTF proteins were aligned using Clustal W. Subsequently, a phylogenetic tree was generated with MEGA 7 employing the neighbor-joining (NJ) method, supported by 1000 bootstrap replicates. For better visualization, the tree was characterized into five subfamilies, each distinguished by different colors and appropriately labeled.

To discover the phylogenetic relationships of trihelix transcription factors (TTFs), a neighbor-joining (NJ) phylogenetic tree was built with *P. trichocarpa*, *A. thaliana*, and *P. euphratica* in order to determine the classes of the *PeuTTF* proteins. Five subgroups of the *PeuTTF* genes were identified: GT-1, GT-γ, GT-2, SH4, and SIP1, as depicted in Figure 1 and Supplementary Table S1(a&b). This classification is comparable to previous studies on *P. trichocarpa* and *Arabidopsis*. All 35 of the PeuTTF proteins were distributed across these subfamilies. The GT-2 clade was the leading subfamily, comprising 15 trihelix proteins, while the SH4 subgroup was the least, with just 1 member. GT-γ was the only clade that was not distributed with any of these gene family members. These distribution tendencies were comparable to those found in *P. trichocarpa* and *Arabidopsis* [25]. The results showed that the dispersal of *PeuTTF* genes was not uniform among the five categories.

Furthermore, the phylogenetic tree demonstrated both orthologous and paralogous links between these three species (Figure 1 and Table 1). *P. euphratica* was found to harbor 7 pairs of paralogous proteins, with strong bootstrap support of 100, 100, 100, 100, 100, 100, 100, 100, 100, 100, 100, 100, 100, and 99, respectively. These pairs included *PeuTTF14* and *PeuTTF17*, *PeuTTF15* and *PeuTTF18*, *PeuTTFT11* and *PeuTTF12*, *PeuTTFT6* and *PeuTTF20*, *PeuTTF4* and *PeuTTF24*, *PeuTTF5* and *PeuTTF31*, *PeuTTF2* and *PeuTTF26*, *PeuTTF1* and *PeuTTF25*, *PeuTTF33* and *PeuTTF34*, *PeuTTFT10* and *PeuTTF28*, *PeuTTFT7* and *PeuTTF8*, *PeuTTF9* and *PeuTTF29*, and *PeuTTF22* and *PeuTTF35*. Moreover, the phylogenetic tree’s bottom placement of groups GT-1 and GT-2 supports the theory that GT1 and GT2 split apart early in the history of land plants. From the two species, we found orthologous and paralogous genes, which are shown in Table 1. In *P. euphratica* and *P. trichocarpa*, 14 and 29 pairs of orthologous genes, respectively, were found; however, no orthologs were found between *P. euphratica* and *A. thaliana*. In light of this, we deduced that the TTF genes of the two poplars had closer ties with each other than with the dicotyledons—*Arabidopsis*—which was dependent on the evolutionary link between these two poplar species. Based on this analysis, 14 pairs of paralogous genes were identified, all of which had strong bootstrap support (>90%).

**Figure 2:**
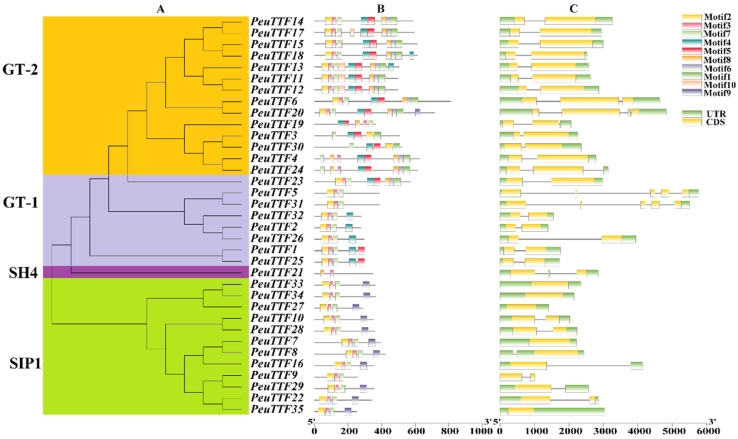
Analysis of the phylogenetic tree, motifs, and gene structure. (**A**) A phylogenetic tree was generated with MEGA 7 employing the neighbor-joining (NJ) method, supported by 1000 bootstrap replicates. (**B**) The conserved motifs in TTF proteins were visualized using motif discovery techniques (MEME). The 10-motif composition model for the entire amino acid sequences of PeuTTF was generated via MEME.XML with the aid of TBtools software (Toolbox for Biologists v1.130). Different motifs are illustrated using boxes of various colors. (**C**) Examination of the exon/intron structure of TTF genes, where green and yellow boxes represent exons, while gray lines denote introns.

Putative conserved motifs predicted by MEME showed the diversity of the *P. euphratica* trihelix genes (Figure 2B). In this prediction, 10 distinct motifs were acknowledged. Notably, motif 1 only existed in GT-2 and GT-1 gene categories, although motif 2 and motif 3 were found in the majority of genes in *PeuTTFs*. On the other hand, GTγ and SIP1 subfamilies had similar motifs, whereas motif 1 and motif 2 were almost in all TTF family members in *P. trichocarpa* [25]. Figure 2C displays the furthermost closely related trihelix members in the identical subfamilies’ joint comparable gene structures in relations to intron statistics or exon lengths. The phylogenetic study was supported by the similarity in gene structures. Remarkably, most genes had only one or two exons, excluding *PeuTTF5* and *PeuTTF31* in the GT-1 subfamily (Figure 2C). The study revealed highly conserved TTF protein sequences within poplar subgroups, with common motif compositions among closely related members, proposing functional relationships among trihelix proteins within the identical subgroup.

**Figure 3:**
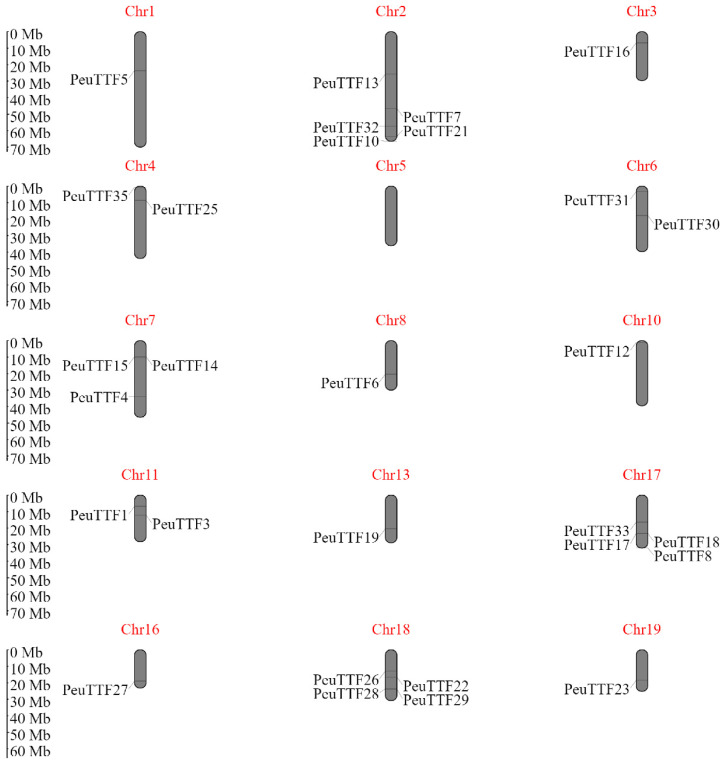
The chromosomal positions of TTF genes in Euphrates poplar species were investigated. In total, 35 *PeuTTF* genes were identified, distributed on chromosomes 1, 2, 3, 4, 6, 7, 8, 10, 11, 13, 16, 17, 18, and 19. The chromosome numbers for each gene are displayed at the top and bottom of their respective chromosomes.

Trihelix genes were substantially plotted on 19 linkage groups in *PeuTTF* genes. Chromosome 2 contains 5 genes, which was the uppermost total, followed by 4 genes on chromosomes 17 and 18. Dissimilarly, no genes were positioned on chromosomes 5, 9, 12, 14, or 15 (Figure 3). Thus, our study examined the relationship between *P. trichocarpa* trihelix genes and duplicated blocks within the *Populus* genome, revealing that 44 genes were located in duplicated regions, with 30 genes present in both copies and 12 genes found outside the duplicated blocks [25].

### 2.3. Forecast Inquiry of Cis-Acting Elements of TTF Family in Poplar Species

Trihelix transcription factors (TTFs) are a family of regulatory proteins characterized by their unique DNA-binding domain formed by trihelix motifs. In *P. euphratica*, an important tree species known for its adaptability to arid regions, understanding the cis-acting elements in the promoter regions of TTF genes can provide insights into their regulatory mechanisms and functional roles in stress responses. The cis-acting elements of the *PeuTTF* gene promoters were identified by querying the DNA sequences located 1500-bp upstream and downstream of the *PeuTTF* genes against the PlantCARE database, aiming to elucidate the regulatory mechanisms governing TTF gene expression in response to biotic and abiotic stressors. Numerous cis-elements relevant to phytohormones and the abiotic stress response were found in the promoter regions of the *P. euphratica* trihelix genes (Supplementary Tables S4 and S5). Transcription factors are regulated transcriptionally in large part by cis-regulatory elements (CREs). Out of all the *PeuTTFs*, a total of 48 CREs were discovered. All detected CREs were grouped into five major categories according to how they were involved in biological functions, as illustrated in Supplementary Table S5. There were no consistent alterations when comparing the anticipated cis-acting elements amongst the subfamily members, indicating that the *PeuTTF* family may have a variety of roles in plants. MYC, GC-motif, anaerobic response element (ARE), wound-response element 3 (WRE3), and MYB are among the CREs linked to both biotic and abiotic stress that originated in the majority of *PeuTTFs*. Comparably, the majority of *PeuTTF* promoters contained a large number of CREs, like G-box (CACGTG), which is involved in light responsiveness and important for photoregulation; ABRE (ACGTG), which is associated with abscisic acid responsiveness and significant for stress responses; DRE (CCGAC), which is responsive to drought and cold, indicating stress adaptation; MBS (CAACTG), which is a MYB-binding site involved in drought inducibility; and LTR (AAAG), which is related to low-temperature responsiveness.

**Figure 4:**
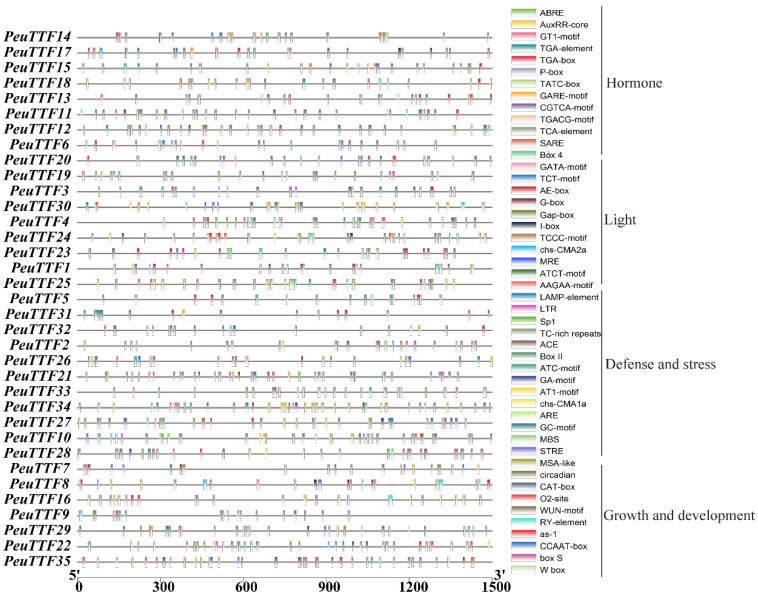
Cis-acting promoter elements in 35 *PeuTTF* genes. Different cis-acting elements are shown in the schematic depiction of the 1500 bp promoter region upstream of the *PeuTTF* genes. Based on the promoter region, they divided it into four types of crucial promoters, including phytohormones, light, defense and stress, and growth and development. These elements are each indicated by boxes of a different color.

The TTF genes contained four classes of cis-acting elements. The first class includes elements that respond to phytohormones, such as ABRE, GARE motif, P-box, TATC, and TGACG. These elements play a fundamental role in plant defense signaling, such as pathogen resistance. The second class includes elements that are anaerobically induced, anoxia-specific inducible, drought inducible, and defense and stress responsive. The third class includes plant growth and development elements, such as endosperm expression cis-acting regulatory elements, meristem expression cis-acting regulatory elements, and circadian control cis-acting elements. The final class includes light-responsive elements, like AE-Box, ACE, G-box, and GT1-motif (Figure 4). Variability in the existence and arrangement of cis-acting elements across different TTF gene promoters indicates potential functional diversification. In addition, our analysis showed that promoter regions contained cis-elements stimulated by hormones, like SA, methyl jasmonate, ABA, gibberellins, and auxin. This suggests that hormones, assisted by cis-elements connected with auxin, gibberellins, ABA, and SA, play a decisive role in plant development [41]. Furthermore, in both normal and drought conditions, the crosstalk between ABA and auxin signaling is essential for controlling root growth and development. A phytohormone known as the “stress hormone,” abscisic acid (ABA), modifies a number of morphological, physiological, biochemical, and molecular processes in plant root tissues to control a poplar’s ability to withstand drought [42]. ABA interacts with both MeJA and SA signaling pathways to battle environmental stress [43]. Auxin, a plant hormone, is crucial in root development, affecting cell fate, meristem initiation, emergence, and elongation [44]. Roots adapt to changing abiotic conditions, including water availability, resulting in hydropatterning and xerobranching, where lateral roots are positioned in high water content areas [45]. Moreover, the interaction between the ABRE and DRE cis-elements and the associated transcription factors (TFs) is essential to comprehending transcriptional gene control and the stress response of plant tissue [46]. Numerous cis-elements associated with stress responses exist in the promoter sequences of *PeuTTF* genes, indicating possible relationships between these cis-elements and transcription factors that respond to abiotic stress. Hence, pathogen tolerance may be predisposed by definite TTF genes in poplar species [25]. Thus, our analysis revealed that the TTF gene family in *P. euphratica* could promote adaptation to its environment due to the accumulation of various cis-acting elements in their promoters. The prevalence of elements responsive to abiotic stressors, such as drought, phytohormones, and light, suggests that these transcription factors play imperative roles in the survival of this species under challenging conditions.

### 2.4. Strong Purifying Selection in TTF Gene Family in P. euphratica

We examined the duplicate procedures in the genome of *P. euphratica* (Table 1) in order to show the expansion pattern of TTF genes. *PeuTTFs* underwent multiple duplication events during their evolutionary stages. Ka and Ks replacement rates for every duplicate pair of *PeuTTF* genes were computed in order to examine the selection pressure that existed during the evolution of the *PeuTTF* family (as illustrated in Table 1). Then, using the formula T = Ks/2λ [47], the timing of duplication occurrences was determined (Table 1). According to these findings, 1 pair (*PeuTTF22* and *PeuTTF35*) out of the 14 pairs of paralogous genes had Ka/Ks rates less than 1, and the corresponding timing of the duplication events (about 189 Mya) indicated that some TTF genes had experienced strong negative selection, suggesting that most TTF genes are developing more gradually. Respectively duplicated pairs’ Ka/Ks ratios were significantly less than 1, suggesting that these genes were chosen for purification. The replicated pairs in *P. euphratica* had somewhat greater Ka/Ks ratios than those in *P. trichocarpa*, suggesting that *P. euphratica*’s TTF genes may be more conserved in poplar species.

### 2.5. Synteny and Evolutionary Analysis of PeuTTF Genes

**Figure 5:**
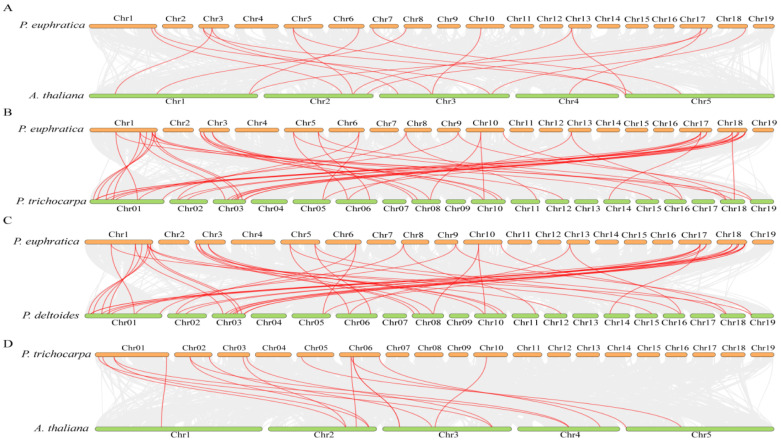
Synteny analysis of PeuTTF and other species. TTF gene collinearity between *P. euphratica* and three representative plants (*A. thaliana*, *P. trichocarpa*, and *P. deltoides*) was analyzed using comparative genomics. (**A**–**C**) letters indicate the synteny collinearity comparison between *P. euphratica* and three other species; (**D**) illustrates the synteny analysis of *P. trichocarpa* and *A. thaliana*. While the red lines in the background indicate homozygous TTF gene pairs, the gray lines in the background indicate blocks of collinearity within *P. euphratica* and the indicated plants.

Using MCScanX analysis to study a gene family aims to provide an image of the genomic distribution and syntenic relationships between the genes on different chromosomes and in different species. A synteny exploration of TTFs between *P. euphratica* and the other three species (*A. thaliana*, *P. trichocarpa*, and *P. deltoides*) was conducted in order to gain further insight into the evolutionary processes of *PeuTTFs* in *Populus*. The following pairings of *P. euphratica* were found: 9 pairs with *A. thaliana* (A), 29 pairs with *P. trichocarpa* (B), 27 pairs with *P. deltoides* (C), and 17 pairs between *P. euphratica* and *A. thaliana* (E). Amongst the TTF synteny segments identified, the most significant alignments were observed between *P. euphratica* and *P. trichocarpa* (Figure 5 and Supplementary Table S7). The results demonstrated that compared to TTF synteny between *P. euphratica* and *A. thaliana*, TTF synteny within the poplar species was further conservative.

These results suggest that the TTF gene family is neither significantly expanding nor contracting throughout the Salicaceae. Instead, it is mostly conserved. In particular, eight pairs of *PeuTTF* genes revealed synteny links between *P. euphratica* and the other two Salicaceae species. This suggests that these TTFs have synteny sections that likely predate the original separation. The preservation of synteny segments containing TTF could have been facilitated by the entire genome duplication of the Salicaceae family. *PeuTTF21* (POPULUS_EUPHRATICA_03745.t2) correlated with four *P. trichocarpa* gene pairs, whereas *PeuTTF5* (POPULUS_EUPHRATICA_24666.t1) was shown to be syntenic with three *A. thaliana* gene pairs. *P. trichocarpa* demonstrated a remarkable degree of 29 orthologous pairs with the reference genomes, followed by *P. deltoides* with 27 orthologous gene pairs spread across chromosomes 1, 6, 8, 9, 10, 13, 17, and 18 and *A. thaliana* with 9 orthologous gene pairs on chromosomes 1, 6, 7, 8, 10, 13, and 17 (Supplementary Table S7). These results demonstrate the substantial degree of orthology that the *PeuTTF* genes demonstrated with the reference genomes. Seventeen *PtrTTFs* were linked to sixteen syntenic gene pairs each, according to additional analysis of the syntenic linkages between *P. trichocarpa* and *A. thaliana* TTF genes (Figure 5). None of the *PtrTTF* members were found to be connected to any syntenic gene pairs.

### 2.6. Examination of the Expression of PeuTTF Genes Utilizing RT-qPCR Analysis

**Figure 6:**
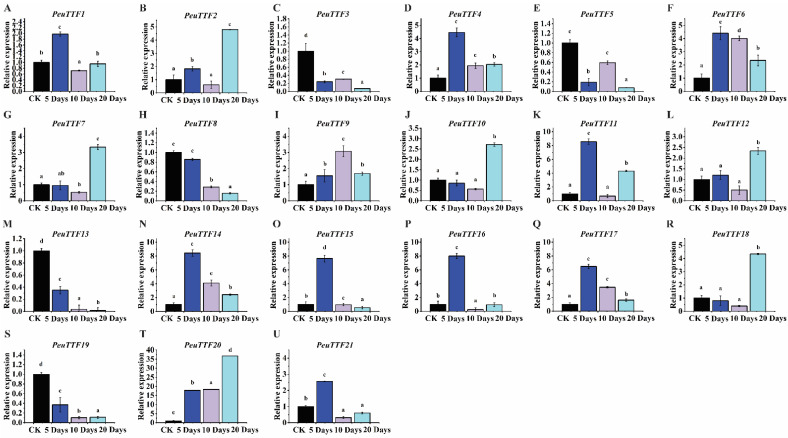
Expression analysis of trihelix genes in leaves under drought stress by RT-qPCR. The expression of the 21 selected *PeuTTF* genes, *PeuTTF1* to *PeuTTF21* (**A**–**U**), respectively, in *P. euphratica* leaves under drought stress conditions. The x-axis shows time after the onset of stress treatments. The standard deviations of the data are displayed by the error bars. Three biological replicates were used. Four types of drought were examined along with the related soil volumetric water content (soil-VWC): mild drought (31 ± 1% soil-VWC), moderate drought (21 ± 1% soil-VWC), severe drought (11 ± 1% soil-VWC), and control (41 ± 1% soil-VWC). In the control group, the relative expression was set to 1. Using Duncan’s test and one-way ANOVA, the relative expression of *PeuTTFs* was compared to the control. Information was displayed as the mean ± standard error (SE). Three replicates’ standard deviations are shown by error bars. The stress treatment groups indicated by the letters (a, b, c, and d) showed a significant difference. To determine the *p*-values (*p* ≤ 0.05), the least significant difference (LSD) test was employed.

To evaluate the validation of stress-related expression levels in *PeuTTF* genes, we conducted RT-qPCR tests on plants subjected to drought and salt stress for various durations: 0 h (control), 120 h (5 days), 240 h (10 days), and 480 h (20 days) for drought, and 0 h (control), 24 h, 48 h, and 96 h for salt stress. This study aimed to explore the expression levels of individual *P. euphratica* trihelix genes. Both selected candidate genes used in this study, *P. euphratica* actin (as illustrate in Supplementary Table S5), were utilized as a positive control to validate the effectiveness of the treatments during the PCR process. Several candidates from the *PeuTTF* gene family displayed either up-regulation or down-regulation. The trihelix genes in *P. euphratica* respond variably to drought and salt stress. In particular, 21 selected *PeuTTF* genes from various subfamilies had varying expression levels when subjected to drought stress, while 5 *PeuTTF* genes changed when exposed to salt stress. Based on their cis-acting regulatory elements and their responses to various treatments, these genes were selected as study subjects. Most *PeuTTF* genes were down-regulated across the three tissues (leaves, stems, and roots) following drought treatments, with the lowest expression occurring after five days. This pattern included genes such as *PeuTTF1*, *-4*, *-6*, *-11*, *-14*, *-15*, *-16*, *-17*, and *-21* in the leaves (refer to Figure 6). The remaining 12 genes exhibited significant differences in their expression patterns when exposed to abiotic stress in leaf organs/tissues. Among these, three *PeuTTF* genes—*PeuTTF6*, *PeuTTF9*, and *PeuTTF20*—were particularly up-regulated in response to drought stress compared to the control. In contrast, the expression levels of *PeuTTF2*, *PeuTTF7*, *PeuTTF12*, and *PeuTTF18* increased and peaked after 20 days of drought treatment. On the other hand, genes, such as *PeuTTF8*, *PeuTTF13*, and *PeuTTF19*, exhibited a consistent reduction in expression, indicating their importance in enduring stress conditions. This continuous drop in expression could play a vital role in preserving cell hydration, regulating stomatal conductance, facilitating chloroplast signaling, and boosting antioxidant defenses. These findings suggest that these genes may play critical roles in the response to drought stress in the leaves of poplar species.

**Figure 7:**
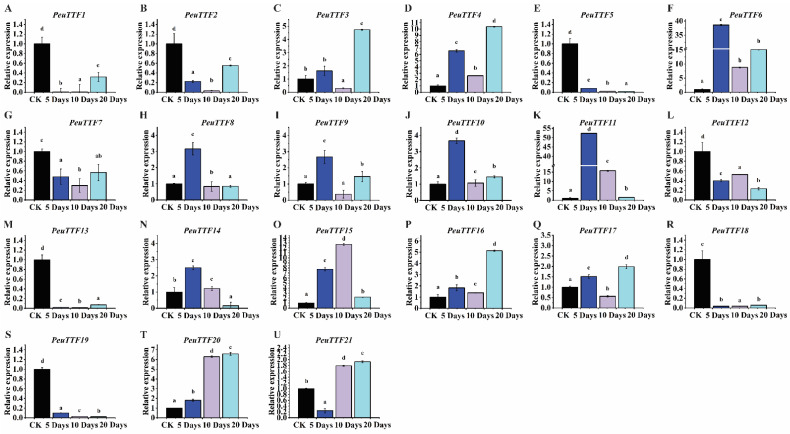
Expression analysis of trihelix genes in stems under drought stress by RT-qPCR. The expression of the 21 selected *PeuTTF* genes, *PeuTTF1* to *PeuTTF21* (**A**–**U**), respectively, in *P. euphratica* stems under drought stress conditions. The x-axis represents the time elapsed after the initiation of stress treatments, with error bars indicating the standard deviations of the data. The study included three biological replicates. Four drought conditions were analyzed, each associated with specific soil volumetric water content (soil-VWC): mild drought (31 ± 1% soil-VWC), moderate drought (21 ± 1% soil-VWC), severe drought (11 ± 1% soil-VWC), and a control group (41 ± 1% soil-VWC). In the control condition, the relative expression was established as 1. The relative expression of *PeuTTFs* was compared to the control using Duncan’s test and one-way ANOVA. The stress treatment groups, labeled with letters (a, b, c, and d), exhibited significant differences. The least significant differences (LSDs) test was utilized to determine the *p*-values (*p* ≤ 0.05).

Most TTF genes exhibited an upward trend in expression after 20 days of drought stress in the stem organs of *P. euphratica*, including *PeuTTF3*, *PeuTTF4*, *PeuTTF6*, *PeuTTF9*, *PeuTTF15*, *PeuTTF16*, *PeuTTF17*, *PeuTTF20*, and *PeuTTF21*, when compared to the control group (CK). In contrast, the expression levels of *PeuTTF6*, *PeuTTF8*, *PeuTTF9*, *PeuTTF10*, *PeuTTF11*, and *PeuTTF14* showed notable decreases after 5 days of treatment in the stems. Furthermore, the expression of *PeuTTF13*, *PeuTTF18*, and *PeuTTF19* significantly declined in the stems after 5 days, highlighting their significance under prolonged stress conditions. This significant reduction in expression may play a key role in maintaining cell hydration and strengthening the antioxidant defenses. Notably, *PeuTTF16*, *PeuTTF20*, and *PeuTTF21* were the most significantly up-regulated in the stem organs (as illustrated in Figure 7). It is possible that *P. euphratica* exhibits enhanced tissue tolerance due to the higher level of expression in stems. These findings suggest that poplar species may exhibit a significant response in their stem organs.

**Figure 8:**
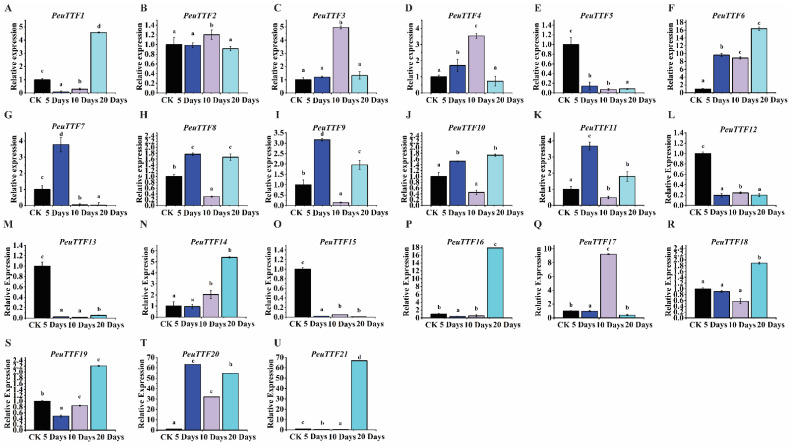
Expression analysis of trihelix genes in roots under drought stress by RT-qPCR. The expression of the 21 selected *PeuTTF* genes, *PeuTTF1* to *PeuTTF21* (**A**–**U**), respectively, in *P. euphratica* roots under drought stress conditions. The x-axis shows time after the onset of stress treatments. The standard deviations of the data are displayed by the error bars. Three biological replicates were used. Four types of drought were examined along with the related soil volumetric water content (soil-VWC): mild drought (31 ± 1% soil-VWC), moderate drought (21 ± 1% soil-VWC), severe drought (11 ± 1% soil-VWC), and control (41 ± 1% soil-VWC). In the control group, the relative expression was set to 1. Using Duncan’s test and one-way ANOVA, the relative expression of *PeuTTFs* was compared to the control. The stress treatment groups indicated by the letters (a, b, c, and d) showed a significant difference.

Moreover, considering the essential role of roots in supporting and nourishing the entire *P. euphratica* plant, we aimed to analyze their expression patterns under drought stress. The expression of *PeuTTF* was notably abundant in the roots and significantly enhanced in response to water deficiency across various stress treatments when compared to the control (CK). After undergoing drought stress treatment, the expression levels of the *PeuTTF7*, *PeuTTF8*, and *PeuTTF9* genes in roots exhibited a general trend of increasing initially and then decreasing, peaking at 5 days. Conversely, the *PeuTTF5-13-15* genes were down-regulated following drought treatments, reaching their lowest expression at control (CK) levels. Additionally, after 20 days, the expression of the *PeuTTF1-6-10-16-18-21* genes showed an increase, achieving high levels in the roots. The *PeuTTF3*, *PeuTTF4*, and *PeuTTF17* genes were meaningfully up-regulated, with their highest expression detected at 10 days of drought stress treatment. In contrast, *PeuTTF2*, *PeuTTF8*, *PeuTTF10*, and *PeuTTF20* displayed an upward trend, peaking in expression at 10 days (as shown in Figure 8). Notably, the expression of the *PeuTTF14* gene consistently increased, reaching its peak at 20 days. This suggests that TTF expression may be a response to drought stress in root organs.

**Figure 9:**
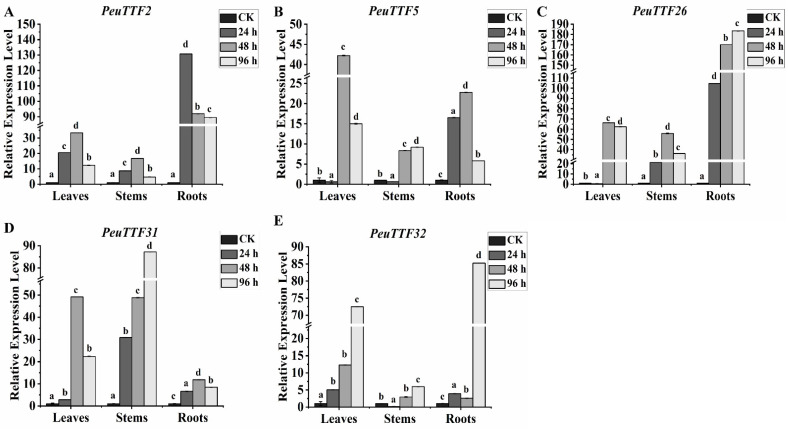
Expression analysis of trihelix genes in leaves, stems, and roots under salt stress by RT-qPCR. The expression of the five selected *PeuTTF* genes, *PeuTTF2*, *PeuTTF5*, *PeuTTF26*, *PeuTTF31*, and *PeuTTF32* (**A**–**E**), respectively, in *P. euphratica* leaves, stems, and roots under salt stress conditions. The five chosen *PeuTTF* genes’ response to NaCl treatment at 24 h, 48 h, and 96 h. *PeuTTF* expression assessments in various tissues, such as (**A**) leaves, (**B**) stems, and (**C**) roots. The control’s relative expression was set to 1. Duncan’s test and one-way ANOVA were used to compare the *PeuTTFs* expression relative to the control. The mean ± standard error (SE) was used to display the data. The standard deviations of three replicates are shown by error bars. Significant differences were indicated by the letters a, b, c, and d, and *p*-values (*p* ≤ 0.05) were determined using the least significant difference (LSD) test. In this investigation, three biological repetitions were used.

In addition, TTF gene expression levels were examined in *P. euphratica* following multiple salt stress exposures (Figure 9). According to the findings, salt stress markedly raised the expression levels of *PeuTTF2*, *PeuTTF5*, *PeuTTF26*, *PeuTTF31*, and *PeuTTF32*. After being exposed to salt stress for 24 h, *PeuTTF5* dramatically increased in roots but did not change in leaves or stems. In contrast to the control, *PeuTTF26* dramatically dropped in leaves after 24 h of salt exposure. At 24 h following salt stress, stems exhibited a substantial drop in *PeuTTF32* in comparison to the control. Remarkably, following salt stress, leaves and roots exhibited considerably higher expression levels of *PeuTTF2*, *PeuTTF31*, and *PeuTTF32* than the control. It is hypothesized that these changes are closely connected to the plant’s methods for adapting to saline environments, possibly by adjusting the osmotic balance and maintaining ion homeostasis levels. These findings demonstrated that *P. euphratica* TTF members could favorably respond to salt stress.

Euphrates poplar is an essential woody tree species within an economic ecosystem, significantly contributing to the fight against desertification. However, a lack of sustainable research aimed at preserving its vitality in arid and semi-arid regions could leave it vulnerable to both biotic and abiotic stressors, such as pathogens and salinity. These salt levels may rise due to poor irrigation practices and the use of saline-contaminated water, leading to an increased concentration of soluble salts in the soil. Irrigation salinity can be caused by salt remaining in the soil after plants take up water or it evaporates. Salt (NaCl) exerts various stressors on plants, leading to significant alterations in osmotic water balance and an elevated concentration of harmful ions within cells. This can result in membrane destabilization, ion toxicity, and oxidative stress. Salinity affects plants in multiple ways, including alterations in photosynthetic parameters, disruptions in nitrogen uptake, stunted growth, cessation of reproduction, and toxicity from harmful ions, such as chloride, that can lead to plant death [48]. Given these challenges, it is decisive to categorize and address the key genes that enhance salt tolerance, which could improve the ability of species to thrive in saline environments. In this regard, we propose a schematic model to explore the salinity effects on this woody tree through important transcription factors, which will contribute to bolstering its salt tolerance.

**Figure 10:**
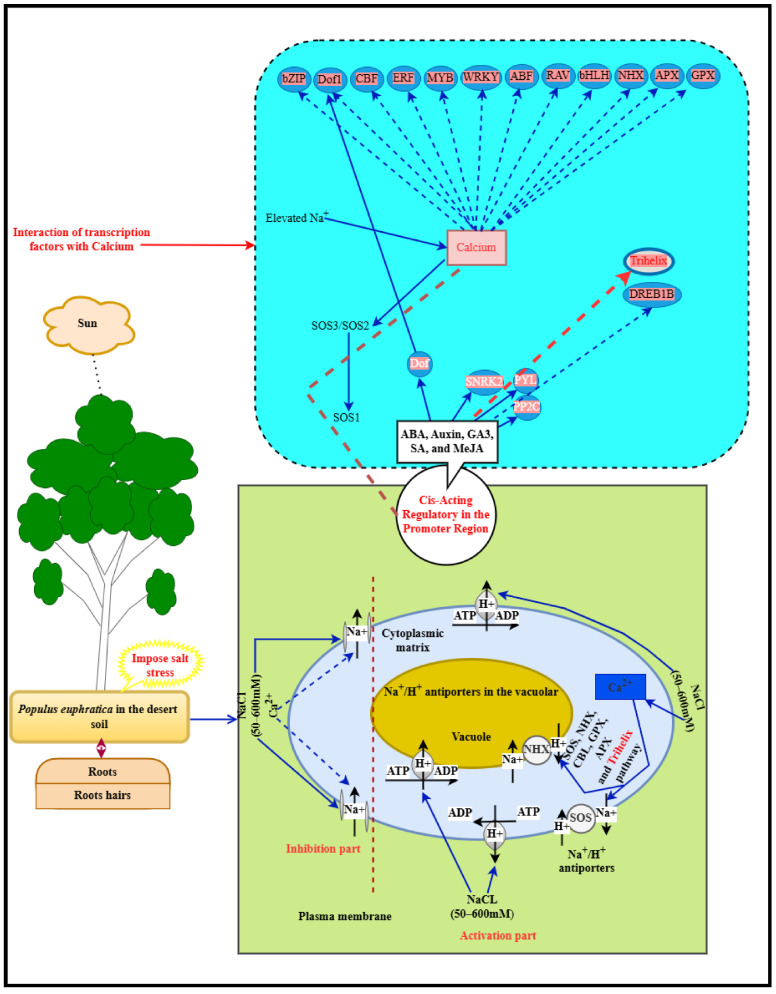
The suggested schematic model for the effect of saline stress in *P. euphratica*: Synopsis of salinity effects on the cytoplasmic matrix and vacuole of woody plants. Salinity induces complex regulation and compartmentalization of ions within woody plants, leading to the synthesis of compatible solutes, specific proteins, and various metabolites. Interactions involving cis-acting elements facilitate physiological and molecular responses mediated by transcription factors with calcium ions (Ca^2^⁺). These responses include the activation of reactive oxygen species (ROS) scavengers, such as catalases, superoxide dismutase, and peroxidases, as well as the modulation of hormone-related compounds, including abscisic acid, auxins, salicylic acid, gibberellic acid, and methyl jasmonate. A specific group of transcription factors is pivotal in regulating these salinity responses, initiating metabolic and molecular networks that enhance salt tolerance. Thus, a comprehensive understanding of the metabolic pathways and molecular networks involved is essential for developing strategies to improve salt tolerance in *P. euphratica* species in arid environments.

Salinity significantly impacts the growth and productivity of *P. euphratica*, a key desert plant found in the arid regions of Central Asia. Leaf traits show that economic and hydraulic characteristics are stable under moderate salt stress but exhibit inconsistency at the onset and conclusion of salt exposure [49]. Regarding root growth, salt hinders root development; however, low nitrogen application can stimulate it [16]. Young saplings tend to sequester toxic ions, like Na^+^ and Cl^−^, in their roots to prevent accumulation in the leaves (as demonstrated in Figure 10). Additionally, there are sex differences, as male *P. euphratica* plants demonstrate greater salinity resistance than females, exhibiting higher biomass, net photosynthetic rates, and more efficient use of water and nitrogen [50]. Finally, the presence of *P. euphratica* diminishes as soil salinity increases. To understand how *P. euphratica* responds to salt stress through hormone regulation, we created a comprehensive hormone–transcriptional regulatory network. Plant hormones, such as ABA, Auxin, GA3, SA, and MeJA, play strategic roles in response to salt stress. The ABA-dependent pathway is particularly significant in *P. euphratica*’s response to this stress. The presence of a double negative regulatory system involving ABA response components (PYL, PP2C, and SnRK2) suggests their importance in sensing and transporting ABA in *P. euphratica* under salt stress [51]. Additionally, the trihelix-family member GT-1 is involved in the expression of the Ca^2+^-binding protein calmodulin, which plays a fundamental role in mediating cellular Ca^2+^ signals in response to various stimuli in higher eukaryotes. Genes related to calcium are highly represented in the regulation of salt ion balance. As a significant secondary messenger, Ca^2+^ plays an energetic role in how plants respond to numerous stressors. We subsequently created an integrated signal transduction and transcriptional regulatory network based on existing knowledge of Ca^2+^ signal transduction and regulatory mechanisms. As illustrated in Figure 10, the blue arrows (indicating activation or repression) represent known Ca^2+^ signal transduction pathways in response to salt stress, while other connections link to the trihelix genes identified in our study or transcription factors (TFs) correlated with the established Ca^2+^ signal transduction network. When plants detect increased Na^+^ levels under salt stress, Ca^2+^ and ABA signaling work together to control a series of molecular and physiological responses via the SOS pathway [52]. The interactions among TFs and trihelix genes associated with Ca^2+^ or ABA suggest that these transcription factors encompass numerous genes that participate in balancing sodium and potassium, which is crucial in the response to salt stress, indicating their role in the salt tolerance of poplars. Furthermore, the presence of cis-acting elements in the promoter regions of many genes related to these phytohormones highlights the prospective of trihelix genes to enhance salt tolerance in *P. euphratica*.

## 3. Discussion

### 3.1. Bioinformatics Analysis of TTF Genes in P. euphratica: Evolutionary Relationship, Structure, and Features

There are two major obstacles to plant growth and productivity: a lack of water and highly salinized soil. Plants express a large number of stress-induced genes in order to adapt to these two stresses [53]. Trihelix transcription factors control genes activated by stress by attaching to cis-acting regions in the promoter [54]. Trihelix transcription factors genes are important for the physical growth of plants as well as their responses to the abiotic stressors, including salt and drought. In this work, we found 35 trihelix genes with at least one trihelix domain in *P. euphratica*. The number of trihelix genes that have been identified is smaller than that of *P. trichocarpa* but substantially larger than that of *Arabidopsis* and rice (30 and 31, respectively), indicating the distinctions between herbaceous and woody plants. Tandem and segmental duplications are two examples of the genome expansions and realignments known to be significantly influenced by gene duplications. Our phylogenetic study revealed the presence of 14 paralogous pairs, indicating a high rate of segmental repetition that is advantageous for the evolution of genes.

The 35 genes varied in the number of exons and introns, suggesting that *P. euphratica*’s TTF gene family is diverse (Figure 2C). Nonetheless, the most closely related genes in the same subfamily had similar gene structures with respect to the length of exons and the number of introns. Some genes had distinct exon/intron structures; for instance, some genes from *Arabidopsis* and rice had 16 or more introns [55,56], whereas the *P. euphratica* genes had significantly fewer introns (less than 6), suggesting that the *PeuTTF* genes may have lost introns or that the rice and *Arabidopsis* genes may have gained introns. Some genes had unique exon/intron structures. A maximum of nine genes in both *Arabidopsis* and rice lacked introns, while four genes in *P. euphratica* lacked them. This suggests that either the exons in *P. euphratica* genes had been lost during the evolutionary process, or the exons in the genes from *Arabidopsis* and rice had been gained. All members of the trihelix gene family exhibited motif 1 and motif 3, according to the motif analysis, although certain specific motifs were determined to be exclusive to certain genes. For instance, motif 10 is unique to subgroup GT-1, whereas motif 4 and motif 9 are exclusive to the two subgroups of trihelix genes of GT-1 and SIP1. According to our results, the trihelix gene in *P. euphratica* may benefit from these motifs for functional diversity distinction (Figure 2B).

Gene duplication events are essential for both expanding the genomic content and diversifying the activities of genes since they can lead to the evolution of new functionalities for genes. Compared to rice (31 *OsTTF* genes) and *Arabidopsis* (28 *AtTTF* genes), *P. euphratica* (35 *PeuTTF* genes) has a higher number of TTF genes [21]. *P. euphratica* has the biggest genome and most chromosomes (19) of the 3 species, which probably helped explain why *P. euphratica* possesses more TTF genes than the other 2 species (as seen in Supplementary Table S2). In *P. euphratica*, we found 14 duplicate gene pairs in our investigation (Table 1). Almost all pairs (Supplementary Table S3) exhibited Ka/Ks ratios less than 1, suggesting that these genes had undergone purifying selection. In order to examine the impact of purifying selection on the 14 paralogous pairs, we employed a sliding Ka/Ks window to assess every pair of TTF paralogs (Supplementary Table S3). This evinced unequivocally that the paralogous genes were subject to intense purifying selection. In some locations, eight genes were subject to positive selection, suggesting that *P. euphratica*’s TTF genes were likewise constrained in forward evolution to maintain their stability.

Groups of proteins known as transcription factors bind to the promoter cis-elements of downstream genes with specificity, controlling target gene transcription and expression at specific times and locations as well as stress target gene regulation. These proteins are essential for plant growth and development as well as environmental responses. Numerous studies have shown that the TTF family participates in biotic and abiotic stress. In *P. euphratica*, promoter cis-elements are crucial for both biotic and abiotic stress responses. The majority of the TTF gene promoter regions were found to contain phytohormones as well as cis-elements that respond to biotic and abiotic stress, such as the GARE-motif, TATC-box, P-box, and MBS (Figure 4). A significant number of hormone-related cis-acting elements were discovered in the promoter region, 1500 bp upstream of the trihelix genes, in addition to the typical light-responsive elements. Simultaneously, components related to seed-specific regulation were found, in addition to several cis-acting elements associated with abiotic stressors, like drought and low temperature. These findings imply that *P. euphratica*’s trihelix gene family members might be in charge of several different biological processes.

### 3.2. Expression Pattern Exploration Specifies TTF Genes’ Roles in P. euphratica Development and Stress Response

Drought stress triggers physiological and biochemical responses in plants [57]. The *PeuTTF* gene family is instrumental in enhancing plant tolerance to limited water availability in tree plants, especially in the desertification regions. Roots are upregulated to improve water uptake, stems increase secondary wall formation to strengthen stem integrity, and leaves regulate stomatal closure and accumulate osmoprotectants and antioxidants to maintain cellular integrity. These adaptations are crucial for maintaining plant health during drought [58]. In this study, after withholding water for varying periods of time, we conducted a comprehensive genome-wide analysis on leaves, stems, and roots in *P. euphratica* to investigate the expression pattern features of trihelix gene family members throughout leaf, stem, and root development. The results of the transcriptome study indicated a considerable number of expressed genes in the roots, stems, and leaves of *P. euphratica* across different developmental stages. Furthermore, we focused on analyzing the expression patterns of the trihelix gene family members in various tissues and organs of *P. euphratica*. The results showed that these trihelix gene family members exhibited diverse expression characteristics during the growth of leaves, stems, and roots, suggesting that they may play multiple regulatory roles. It should be noted that the transcriptome study results reveal that some trihelix family members are either not expressed in *P. euphratica* or only express at very high levels after 10 days after treatments of water stress induction. This suggests that these genes may perform regulatory functions in the leaves, stems, and roots of *P. euphratica*. In addition, designing suitable primer pairs is quite challenging because of the abundance of repetitive sequences. Lastly, we determined the tissue-specific expression patterns of these genes by identifying and characterizing the relative expression levels of 21 trihelix gene members, which were successfully confirmed by RT-qPCR analysis. At 20 days following drought stress treatments, for instance, *PeuTTF2*, *PeuTTF7*, *PeuTTF10*, *PeuTTF18*, and *PeuTTF20* were expressed substantially higher in leaves than in stems, but *PeuTTF1*, *PeuTTF6*, *PeuTTF10*, *PeuTTF16*, *PeuTTF18*, and *PeuTTF21* genes were highly expressed in roots. These findings clearly imply that the 21 genes confirmed by RT-qPCR might be crucial to the development of *P. euphratica* organs and tissues under drought stress. Consequently, elevated and highly expressed levels of *PeuTTF6*, *PeuTTF9*, and *PeuTTF20* responded in roots and leaves, suggesting that TTF expression could be a response to drought stress. Furthermore, our investigation revealed that the *P. euphratica* trihelix gene is susceptible to drought and severe salt stress. Research from the past has indicated that salt and drought stress can trigger the expression of the cotton GT-2 gene, Gh-A05G2067 [32]. Rice, tomato, and cotton all had high levels of induction for the GT-1 and GT-2, except GT-γ genes. In *P. euphratica*, our investigation revealed that, with the exception of the GT-γ gene, GT-1 (2, 5, 26, 31, 32), GT-2 (6, 14, 20), SIP1 (9, 10, 16,), and SH4 (21) genes were more susceptible to abiotic stress. It is interesting to note that black cottonwood showed high levels of GT-1, GT-2, and GT-γ gene induction [25]. The *PeuTTF* gene family plays a crucial role within the intricate network of plant responses to abiotic stress, especially regarding drought and salt conditions encountered in arid areas. It is essential to further investigate the specific molecular mechanisms of these genes, their possible interactions with other signaling pathways, and their potential applications in breeding programs aimed at enhancing abiotic stress tolerance in poplar tree species. Gaining a deeper understanding of these regulatory networks will offer valuable insights for future agricultural practices, particularly in light of climate change and rising soil salinity in semi-arid and arid regions.

## 4. Materials and Methods

### 4.1. Plant Materials and Samples Collections

In a greenhouse setting, 2-year-old *P. euphratica* seedlings were grown in plastic pots containing a soil mixture of Inner Mongolian sand, loamy soil, and peat soil in a ratio of 2:2:1 (*v*/*v*). The seedlings were sourced from the Banner region of Inner Mongolia and Alxa regions in China, with 3 or 4 seedlings planted in each 5-L pot [59]. After 3 to 5 months of cultivation under controlled conditions (16 h of light and 8 h of dark, 28 °C day temperature, and relative humidity maintained between 40 and 60%, monitored daily), 4 out of 12 seedlings displaying uniform growth were chosen for each treatment. The young seedlings were irrigated with a 200 mM NaCl solution to induce salt stress over a 2-week period. Following this, the same NaCl concentrations were reapplied. Samples were collected at 0 h (control, untreated), as well as 24, 48, and 96 h post-treatment. For inducing drought stress, the method described by Tang et al. [60] was utilized. Samples weighing 0.2 g were taken from leaves, stems, and roots of 3 plants at the same maturity level for both salt and drought stress experiments. Each sample was immediately frozen in liquid nitrogen and stored in an ultra-low temperature refrigerator at −80 °C.

### 4.2. Identification and Characterization of PeuTTF Gene Family

We used the protein sequences of *A. thaliana* and *P. trichocarpa* as possible queries to compare the trihelix transcription factor family members with the sequences of *P. euphratica*. *Arabidopsis* trihelix transcription factor family member sequences were downloaded from the Plant Transcription Factor Database PlantTFDB (https://planttfdb.gao-lab.org/family.php?sp=Ath&fam=Trihelix, accessed on 6 June 2024). The *P. trichocarpa* trihelix gene sequence was gained from the previous published article [25]. Using queries based on the 34 *Arabidopsis* trihelix transcription factor protein sequences from the Plant Transcription Factor Database (PlantTFDB), we employed BLASTp to search for potential trihelix transcription factor proteins in the *P. euphratica* genome. The E-value cutoff for these queries was 1.0 × 10^−10^. In total, the 57 putative trihelix transcription factors were used and after checking with the pfam database (https://www.ebi.ac.uk/interpro/, accessed on 6 June 2024) through the pfam domain (PF13837, SM00717). After eliminating non-redundant genes, we finally retrieved 35 putative trihelix transcription factor genes in *P. euphratica*. All the trihelix transcription factor candidate proteins were carefully checked and analyzed using the Conserved Domain Database (CDD) of NCBI (https://www.ncbi.nlm.nih.gov/cdd/, accessed on 6 June 2024) and the SMART database (http://smart.embl.de/, accessed on 6 June 2024). We identified 35 proteins in *P. euphratica* and named them according to the gene ID on NCBI (Supplementary Table S2). In this study, the ExPASy tool (https://web.expasy.org/protparam/, accessed on 6 June 2024) was used to show the molecular weight (MW), isoelectric point (pI), and amino acid sequence length of 35 trihelix transcription factor proteins [23]. We used the online DeepLoc 2.0 server (https://services.healthtech.dtu.dk/services/DeepLoc-2.0/, accessed on 6 June 2024) to look into where the PeuTTF proteins were located within cells. We also checked the sequences of the PeuTTF proteins using the BUSCA server (https://busca.biocomp.unibo.it/, accessed on 6 June 2024) to get a better idea of where they might be located within cells [61]. We created a chromosome location figure using an online website (http://mg2c.iask.in/mg2c_v2.1/), which we accessed on 9 June 2024. Based on the locations of homologous in *PtrTTFs* or related genes, the possible chromosomal location of *PeuTTFs* was found and nearly every *PeuTTF* had a corresponding location on the *P. trichocarpa* chromosomes.

### 4.3. Phylogenetic Tree Analysis and Classification of PeuTTF Proteins

The ClustalW tool was used to perform multiple sequence alignment of the AtTTF, PtrTTF, and PeuTTF protein sequences. Then, utilizing MEGA 7.0 software and 1000 bootstrap repeats, an unrooted phylogenetic tree was built using the neighbor-joining (NJ) method [62]. The online iTOL program v6 (https://itol.embl.de/, accessed on 7 June 2024) was used to display and enhance the phylogenetic tree [63].

### 4.4. Motif, Promoters and Gene Structure Analysis in PeuTTF Proteins

MEME (https://meme-suite.org/tools/meme, accessed on 10 June 2024) was used to find the conserved motifs of 35 PeuTTF members, up to a maximum of 10 motifs. TBtools (v1.130) was used to create the gene structure map of the *PeuTTF* genes. Cis-acting regulatory elements were annotated using an online tool (https://bioinformatics.psb.ugent.be/webtools/plantcare/html/, accessed on 5 July 2024).

### 4.5. Synteny Analysis of PeuTTFs

Tbtools identified the *PeuTTF* genes’ physical position inside the *P. euphratica* genome database. To examine the *PeuTTF* gene duplication occurrences, the MCScanX program (University of Georgia, Athens, GA, USA) was utilized with its default parameters. The homology of TTF genes between *P. euphratica* and the other species (*A. thaliana*, *P. deltoides*, and *P. trichocarpa*) was examined using the dual synteny plotter in Tbtools.

### 4.6. RNA Extraction and DNA Isolation

The samples were ground in liquid nitrogen immediately after sampling, and the remaining samples were stored in a −80 °C in an ultra-low temperature refrigerator. Total RNA was then extracted from each of the plant samples of leaves, stems, and roots tissues using the RNeasy Mini Kit (Hilden, Germany), with each sample weighing about 200 mg, and the total RNA’s integrity was assessed via 1% agarose gel electrophoresis (Bio Teke Biotechnology, Beijing, China). We evaluated the quality of the RNA with a TGem spectrophotometer Plus (TIANGEN BIOTECH, Beijing, China) by measuring the A260/A280 ratio. We immediately used 1 mg of total RNA from each sample to synthesize the first-strand cDNA with the FastKing gDNA Dispelling RT SuperMix Kit (TIANGEN BIOTECH, Beijing, China). Specific primer pairs were designed using the NCBI Primer Blast online tool (https://www.ncbi.nlm.nih.gov/tools/primer-blast/, accessed on 20 June 2024), and a comprehensive list of these primers can be found in Supplementary Table S6. The reference genes for *P. euphratica* were selected based on the work of Wang et al. [64,65], as cited in [59].

### 4.7. Separation

The Stratagene Mx3000P equipment (Agilent Technologies, CA, USA) was used to perform quantitative real-time PCR to identify the chemical SYBR Green. RT-qPCR was performed in triplicate for each biological replicate to ensure accuracy. The established reaction system is as follows: A reaction mixture totaling 20 µL was prepared, consisting of 10 µL of 2× SuperReal Premix Plus (Tiangen Biotech, Beijing, China), 0.6 µL of each forward and reverse primer (10 µM), 1 µL of diluted cDNA template, and RNase-free ddH_2_O to complete the volume. The PCR cycling protocol consisted of 40 cycles of 10 s at 95 °C and 30 s at 60 °C, followed by a final hold at 95 °C for 15 min. Melting curve analysis was conducted immediately after PCR to measure fluorescence intensity at each temperature increment starting from 60 °C. The RT-qPCR analysis was performed with *Peuactin* as a standardized internal control reference gene.

### 4.8. Statistical Analysis

To assess gene expression, the relative quantity of templates in each PCR run was determined using the 2^−∆∆CT^ method, which normalized the expression levels of control samples to a value of 1 based on three biological replicates. Following this, we conducted the statistical analysis for the study by using Excel version 2024.

### 4.9. Data Interpretation

The data are expressed as means ± SE from a minimum of three independent replicates in a single representative experiment. The data were analyzed by Duncan’s multiple range tests in the ANOVA program of IBM SPSS Statistics 22 (Armonk, NY, USA), taking *p* < 0.05 with significance levels defined through the LSD test. Then, Origin software version 2020 was used to draw the figures of this study.

## 5. Conclusions

The gene structure, conserved domain and motifs, chromosomal location, duplication patterns, cis-acting elements, and expression pattern levels of the TTF genes were investigated for comprehensive analysis in *P. euphratica*. We employed 35 *PeuTTFs* and categorized into 4 classes via phylogenetic topology analysis. Most *PeuTTFs* are largely homologous to the characterized *PtrTTFs* and *AtTTFs*. Protein–protein interaction network and RT-qPCR analyses revealed that *PeuTTF6*, *PeuTTF9*, and *PeuTTF20* responded in roots and leaves, suggesting that TTF expression could be a response to drought stress. Alternatively, after exposure to salt stress, there was a significant increase in the expression of *PeuTTF2*, *PeuTTF31*, and *PeuTTF32* in both leaves and roots compared to the control group (CK). This suggests that these changes may be linked to the plant’s mechanisms for coping with salinity, potentially by regulating osmotic balance and ion homeostasis. Upcoming research on TTFs should focus on their function in *P. euphratica* under different abiotic stressors to identify adaptive mechanisms. The findings of this study may be limited due to the specific genetic backgrounds of the plant materials used and the controlled environments that do not fully reflect natural conditions. Future investigations should use transgenic methods to alter TTF expression and conduct analyses in various environmental settings to confirm their roles in stress response. Genetic variations in TTFs are important for regulating plant responses to environmental challenges in *P. euphratica*, as they help mediate stress tolerance by influencing key pathways. Silencing or enhancing TTF expression can improve resilience to salt stress and support growth and adaptation. This could benefit breeding programs that aim to develop resilient poplar tree species for drought and soil salinity tolerance in arid regions.

## Figures and Tables

**Table 1:** Ka/Ks examination and predictable divergence time of *PeuTTF* genes.

Replicated Gene Pairs	Ka	Ks	Ka/Ks	MYA
*PeuTTF14-17*	0.114524709	0.247471097	0.462780142	18.86212628
*PeuTTF15-18*	0.089673874	0.281693343	0.318338634	21.47052919
*PeuTTF11-12*	0.000847936	0	NA	0
*PeuTTF6-20*	0.069736978	0.283795253	0.245729896	21.63073577
*PeuTTF3-30*	0.102853092	0.234664222	0.438298992	17.88599255
*PeuTTF4-24*	0.106539194	0.301894748	0.352901782	23.01027042
*PeuTTF1-25*	0.039641622	0.319670325	0.124007824	24.36511621
*PeuTTF5-31*	0.078854071	0.296685262	0.265783581	22.61320592
*PeuTTF2-26*	0.002825527	0	NA	0
*PeuTTF33-34*	0.060780096	0.30245201	0.200957818	23.05274467
*PeuTTF10-28*	0.080032165	0.349479041	0.229004191	26.63712202
*PeuTTF7-8*	0.089227969	0.305773083	0.291811064	23.3058752
*PeuTTF9-29*	0.154997555	0.722954562	0.214394601	55.10324404
*PeuTTF22-35*	0.420684735	2.489813287	0.168962362	189.7723542

## Data Availability

All data supporting results associated with this study can be found within the article and within its Supplementary Materials published online.

## Supplementary Materials

The following supporting information can be downloaded at: https://www.mdpi.com/article/10.3390/plants14050662/s1, Tables S1–S7: Bioinformatics inquiries.
